# A 21-Year Diagnostic Odyssey in TBC1D24-Associated Developmental and Epileptic Encephalopathy With Favorable Response to Corpus Callosotomy: A Case Report

**DOI:** 10.7759/cureus.114608

**Published:** 2026-08-16

**Authors:** Alfredo Pacheco-Abbud, Alan Alberto Pérez-Arzola, Adlen S. Martínez Torres, Luis E Fernández-Garza, Victor G Rojas, Marlene Arbeu-Reyes, Daniela Juárez-Melchor, Jacqueline López-Quecho, Israel Enrique Crisanto-López

**Affiliations:** 1 Neurology, "Dr. José Eleuterio González" University Hospital, Monterrey, MEX; 2 Internal Medicine, General Hospital of Zone No. 2, Mexican Institute of Social Security, Monterrey, MEX; 3 Neurology, Movement Disorders and Neurodegenerative Diseases, Mexican Social Security Institute, Pachuca, MEX; 4 University Center for Health Sciences, University of Guadalajara, Guadalajara, MEX; 5 Medical Genetics, General Hospital Zone No. 20, Mexican Social Security Institute, Puebla, MEX; 6 Internal Medicine, Institute for Social Security and Services for State Workers, Puebla, MEX; 7 Reproductive Biology Laboratory, Mexican Social Security Institute, Puebla, MEX; 8 Medicine, Meritorious Autonomous University of Puebla, Puebla, MEX

**Keywords:** genetic counseling, seizures, speech delay, status epilepticus, whole exome sequencing

## Abstract

Developmental and epileptic encephalopathies (DEEs) are one of the most important causes of seizures occurring during the early years of life. Establishing an etiologic diagnosis remains challenging in many affected individuals. Next-generation sequencing (NGS), such as whole-exome sequencing (WES) or targeted gene-panel sequencing, has therefore become a useful tool for identifying these patients. One of the genes implicated in DEEs is TBC1D24. Recessive pathogenic variants in this gene cause a broad phenotypic spectrum that includes familial infantile myoclonic epilepsy, early infantile epileptic encephalopathy 16, and deafness, onychodystrophy, osteodystrophy, mental retardation, and seizures (DOORS) syndrome, among other disorders. No specific treatment is currently available for DEE caused by TBC1D24 variants. We therefore present the case of a Mexican male patient with a homozygous TBC1D24 variant who underwent a 21-year diagnostic odyssey and showed a favorable response to corpus callosotomy.

A 22-year-old right-handed man was evaluated for refractory convulsive status epilepticus. His family history was notable for an older brother who died at 32 years of age from an undetermined cause and had epilepsy beginning at two months of age, intellectual disability, apparent hemiclonic seizures, and an unspecified visual disorder. The patient had global developmental delay involving the motor, language, and cognitive domains. Since disease onset, he had experienced recurrent episodes of status epilepticus that did not respond adequately despite stepwise adjustments to antiseizure therapy and the use of sedation during periods of greatest ictal activity. Palliative surgical treatment with anterior corpus callosotomy was therefore performed, resulting in a reduction in overall seizure burden, focal seizure frequency, recurrence of status epilepticus, and the need for rescue medication.

Given the history of epileptic encephalopathy, intellectual disability, global developmental delay, drug resistance, recurrent status epilepticus, and a deceased brother with a similar neurological phenotype, genetic sequencing was performed and identified the homozygous TBC1D24 variant c.845C>G (p.Pro282Arg), classified as likely pathogenic. The clinical, neuroimaging, and genetic findings supported a diagnosis of TBC1D24-associated DEE 16 with an autosomal recessive inheritance pattern. A multidisciplinary approach is the cornerstone of follow-up for all patients, particularly those with difficult-to-control clinical manifestations and a family history of similar disorders.

## Introduction

Approximately 40% of seizures occurring during the first years of life are caused by developmental and epileptic encephalopathies (DEEs), a group of disorders characterized by epileptic seizures or epileptiform activity, severe delay or regression of psychomotor development, and cognitive and behavioral impairment, usually of genetic etiology [1]. Although DEEs predominantly begin during childhood, most patients reach adulthood, often without a diagnosis, after a prolonged journey involving physical, emotional, and financial difficulties known as the diagnostic odyssey [2,3].

Establishing a genetic diagnosis can guide the clinical evaluation and influence treatment options. In recent years, next-generation sequencing (NGS) has become a standard diagnostic tool in clinical practice. NGS strategies include whole-genome sequencing (WGS), whole-exome sequencing (WES), and targeted gene-panel sequencing. As an initial diagnostic test, WES provides a diagnosis in approximately 30% to 50% of patients with mild-to-severe DEE [4]. Additionally, clinicians can contribute to the comprehensive evaluation of the patient's phenotype and to determining whether an association with the variant identified through targeted gene panel sequencing is plausible [2].

The human TBC1D24 gene encodes a vesicle-associated protein involved in neuritogenesis, neural crest cell and neuronal migration, neuronal maturation, and neurotransmission [5]. Recessive pathogenic variants can cause a broad spectrum of phenotypes, ranging from a mild form of familial infantile myoclonic epilepsy (FIME) (OMIM #605021) and early infantile epileptic encephalopathy 16 (EIEE16) (OMIM #615338) to DOORS syndrome - deafness, onychodystrophy, osteodystrophy, intellectual disability, and seizures (OMIM #220500) - and a syndromic form of sensorineural hearing loss [6,7].

No specific treatment is currently available for DEE caused by TBC1D24 variants. However, recent evidence suggests that procedures such as corpus callosotomy (a neurosurgical procedure in which the corpus callosum is cut or split, spreading it between the hemispheres) can reduce seizures in genetic DEEs [8]. We therefore present the case of a Mexican male patient with a homozygous TBC1D24 variant who underwent a 21-year diagnostic odyssey and showed substantial seizure reduction following corpus callosotomy as part of multimodal management.

## Case presentation

**Figure 1:**
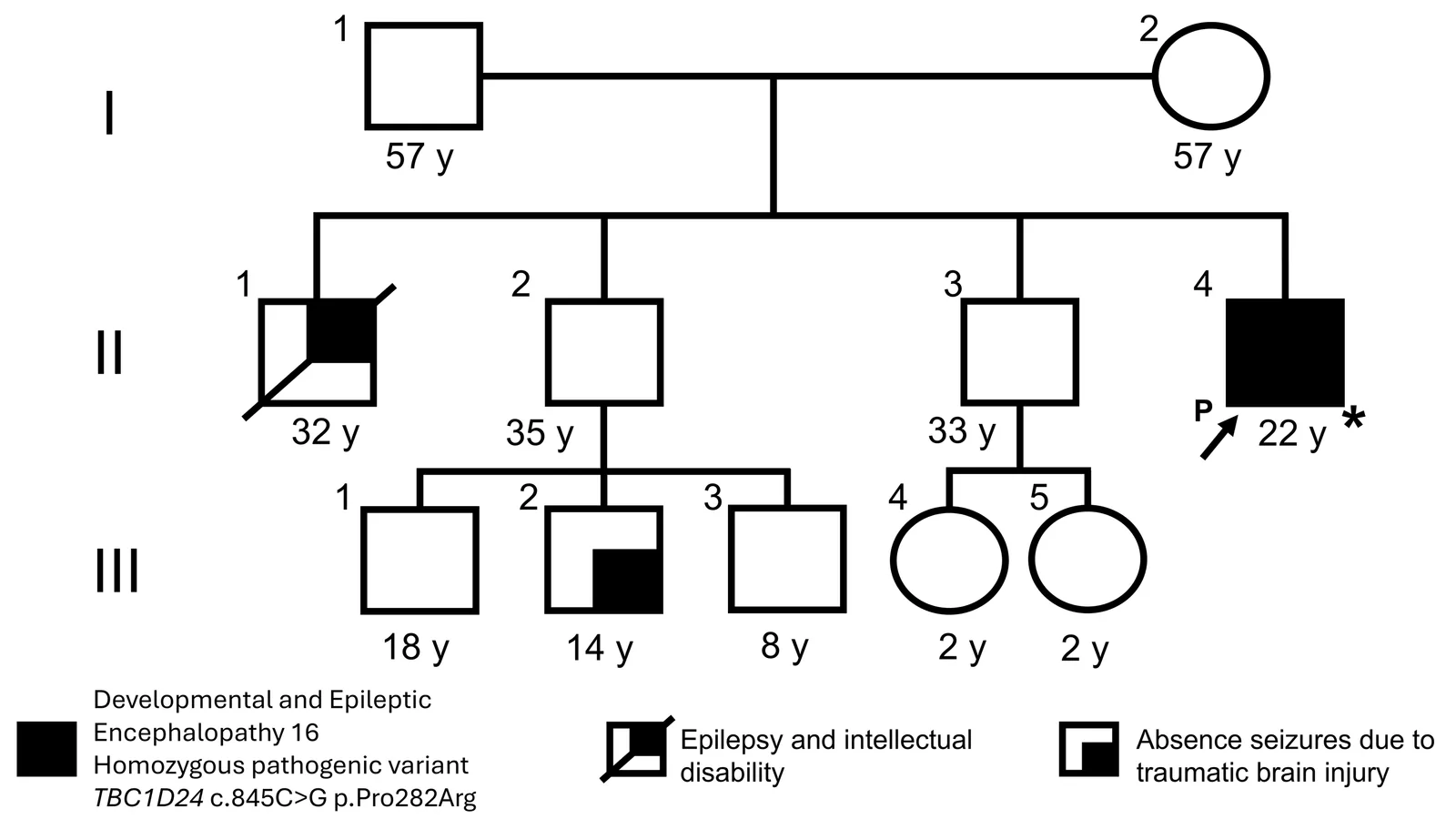
Pedigree of the patient. This image shows a pedigree of a family spanning three generations (I, II, and III), with each individual represented by an Arabic numeral (1, 2, 3, etc.). The blank square (males) and circle (females) represent healthy individuals. The patient is indicated by the arrow (individual 4 in generation II, II-4), in whom the TBC1D24 variant was identified. His brother, individual II-1, had similar clinical features; however, genetic testing could not be performed. Asterisk (*) indicates that the evaluation was documented and the relevant records were reviewed.

As a result of the patient's acute clinical condition, the clinical information was obtained indirectly from a family member. A 22-year-old right-handed man was evaluated for refractory convulsive status epilepticus. His family history was notable for an older brother who died at 32 years of age from an undetermined cause and had epilepsy beginning at two months of age, intellectual disability, apparent hemiclonic seizures, and an unspecified visual disorder. His epilepsy followed a drug-resistant course, with multiple episodes of convulsive status epilepticus during adulthood. Two additional brothers, aged 35 and 33 years, were reported to be clinically healthy. There was no known consanguinity (Figure 1).

The patient was the product of a fourth pregnancy complicated by preeclampsia that was managed conservatively. He was born at term by spontaneous vaginal delivery, with a birth weight of 3,450 g (63rd percentile) and length of 55 cm (96th percentile). Since birth, his mother had observed abnormal eye movements, retrospectively described as nystagmus-like.

The patient had global developmental delay affecting motor, language, and cognitive domains. He achieved unsupported sitting at five months and independent walking at two years of age. His speech in the first year of life was mainly monosyllabic utterances, with a psychological assessment at two years of age and a language disorder diagnosis. He subsequently developed functional language and was able to narrate simple stories. Cognitively, he had marked learning difficulties and never acquired reading or writing skills. Intellectual disability was identified between six and eight years of age, although no quantitative neuropsychological assessment was available. At five years of age, he was diagnosed with attention-deficit/hyperactivity disorder and treated with methylphenidate. He also experienced episodes of irritability that were treated with risperidone, which was subsequently discontinued because of somnolence.

Epilepsy began at two months of age with approximately one seizure per month. The seizures were initially described as motor events with bilateral increased muscle tone. Initial treatment included topiramate, which was discontinued because of intolerance, followed by lamotrigine. From disease onset, he experienced recurrent episodes of status epilepticus, including a first episode at two months of age, with multiple recurrences and impairment of awareness throughout the course of his illness.

The current illness began with upper respiratory tract symptoms that were initially treated with chlorpheniramine. During the following 24 hours, he experienced a focal-to-bilateral tonic-clonic seizure lasting approximately two minutes, followed by a 20-minute postictal period. Cough, somnolence, odynophagia, and lower-extremity pain subsequently developed, together with recurrent convulsive events. One event was treated at home with clonazepam, and another prompted transfer to the emergency department.

On hospital admission, he presented with convulsive status epilepticus characterized by focal tonic-motor seizures involving the right side of the body, predominantly the upper limb. Because of the lack of response to an intravenous benzodiazepine and second-line antiseizure medications, as well as his inability to protect the airway, orotracheal intubation and continuous anesthetic sedation were initiated as third-line management for refractory status epilepticus, together with invasive ventilatory support. The episode occurred in the setting of a respiratory infection, which was considered the main acute precipitating factor.

The initial neurological examination was limited by anesthetic sedation and invasive mechanical ventilation. No spontaneous motor response or withdrawal from painful stimuli was observed. The pupils were symmetric and reactive to light. Primary gaze was dysconjugate, and airway-protective reflexes were preserved. Muscle stretch reflexes were diffusely decreased, plantar responses were bilaterally equivocal, and there were no clinical signs of meningeal irritation.

During hospitalization, the patient had a complicated course characterized by recurrent hemiclonic seizures with orolingual automatisms when anesthetic sedation was reduced. He experienced repeated episodes of refractory status epilepticus requiring reintubation, invasive mechanical ventilation, continuous intravenous anesthetic sedation, and stepwise adjustments to antiseizure polytherapy.

His systemic course was complicated by rhabdomyolysis and acute kidney injury attributed to prolonged convulsive status epilepticus. He also developed respiratory complications associated with bacterial superinfection and invasive mechanical ventilation, resulting in extubation failure and the need for tracheostomy. In addition, he experienced transient septic shock requiring vasopressor support.

**Figure 2:**
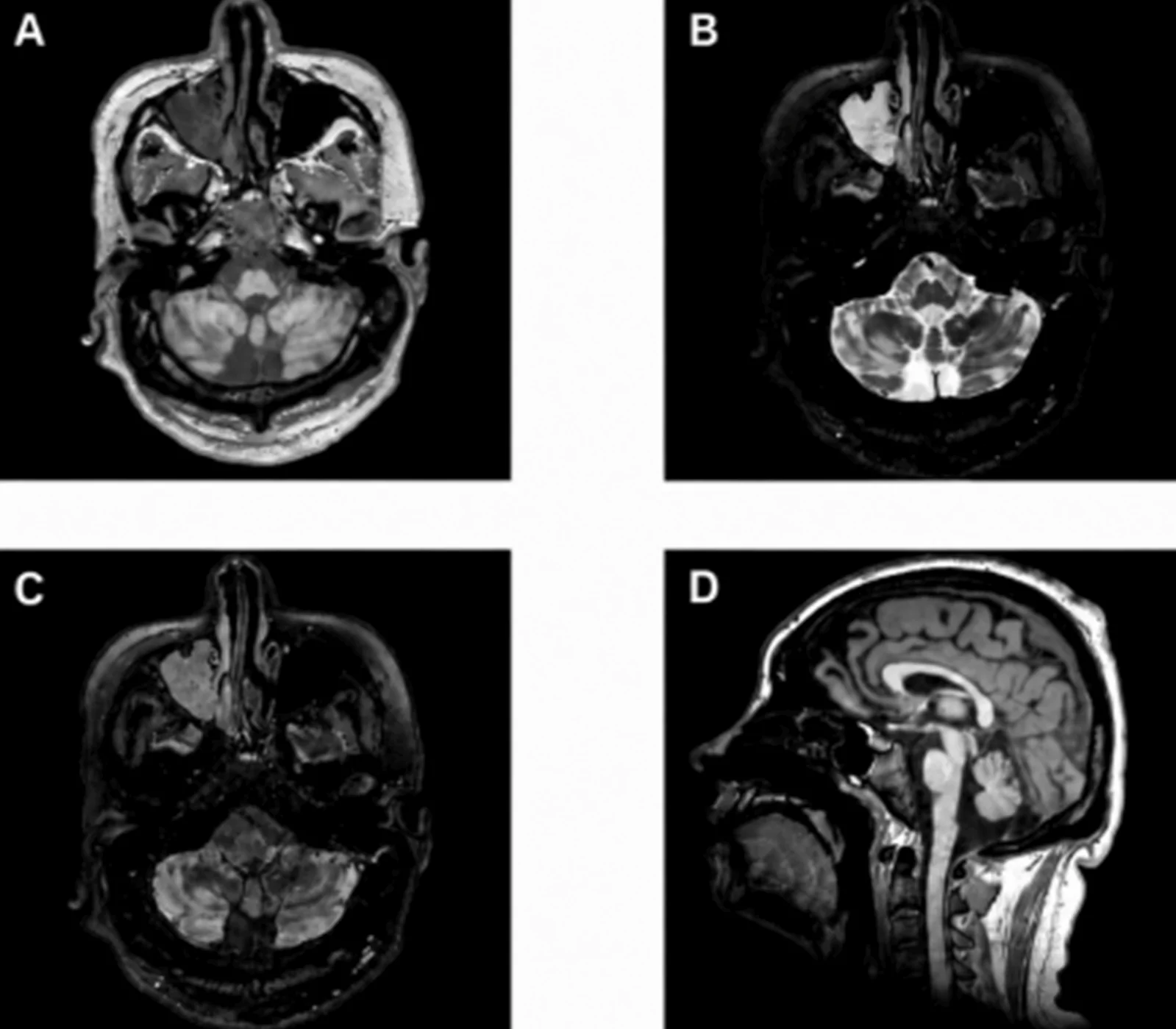
Brain magnetic resonance imaging. (A) Axial T1-weighted image showing a bilateral, relatively symmetric abnormality of the cerebellar hemispheres in the region of the ansiform lobule, characterized by decreased signal intensity. (B) Axial T2-weighted image showing bilateral hyperintensity in the same anatomical distribution and blurring of the gray-white matter differentiation. (C) Axial fluid-attenuated inversion recovery (FLAIR) image confirming bilateral hyperintensity predominantly involving the lateral and posterior cerebellum. (D) Midline sagittal T1-weighted image showing relative sparing of the cerebellar vermis.

Despite stepwise adjustments to antiseizure therapy and the use of sedation during periods of greatest ictal activity, the patient continued to have recurrent focal motor seizures, some progressing to focal-to-bilateral tonic-clonic seizures, as well as further episodes of refractory status epilepticus. Subsequently, after optimization of different antiseizure medication combinations and gradual reduction of continuous sedation, a phase of decreased clinical ictal activity was achieved. In this context, a follow-up electroencephalogram showed persistent epileptiform activity without electroclinical criteria for status epilepticus at the time of recording. Brain magnetic resonance imaging showed bilateral, relatively symmetric abnormalities in the cerebellar hemispheres, predominantly involving the lateral and posterior portions of the ansiform lobule. These abnormalities were characterized by decreased T1-weighted signal, T2-weighted/fluid-attenuated inversion recovery (FLAIR) hyperintensity, and blurring of the gray-white matter differentiation, with relative sparing of the cerebellar vermis on sagittal images (Figure 2).

After continuous sedation was discontinued, neurological examination showed persistence of a chronic ocular abnormality characterized by dysconjugate primary gaze with abduction of the left eye. This abnormality had reportedly been present since childhood and was correctable with oculocephalic maneuvers. No papilledema was observed. Muscle tone was preserved, with motor responses to painful stimuli in all four extremities and mild, asymmetric hyperreflexia, together with subtle pyramidal signs predominantly affecting the left side.

Given the history of epileptic encephalopathy, intellectual disability, global developmental delay, a congenital ocular abnormality, drug resistance, recurrent episodes of status epilepticus, and a deceased brother with a similar neurological phenotype, genetic testing with a targeted epilepsy and ataxia gene panel was requested.

While awaiting the genetic test results, the patient continued to experience multiple daily seizures despite optimized antiseizure polytherapy, including focal motor seizures and focal-to-bilateral tonic-clonic seizures. Because of the high seizure burden, recurrent refractory status epilepticus, and predominance of seizures with bilateral spread, palliative surgical treatment with anterior two-thirds corpus callosotomy was performed.

Molecular testing subsequently identified the homozygous TBC1D24 variant c.845C>G, resulting in the protein change p.Pro282Arg. The variant was classified as likely pathogenic according to the American College of Medical Genetics and Genomics (ACMG) guidelines. Taken together, the clinical (early-onset drug-resistant epilepsy, global developmental delay), neuroimaging (bilateral abnormalities in the cerebellar hemispheres), and genetic findings (likely pathogenic homozygous TBC1D24 variant) supported a diagnosis of TBC1D24-associated DEE 16 with an autosomal recessive inheritance pattern.

Based on the genetic test result and previous reports of patients with TBC1D24 alterations, the antiseizure medication regimen was adjusted to a combination of topiramate, oxcarbazepine, and valproate. Phenobarbital was not considered a maintenance treatment option because of a previous episode of respiratory suppression associated with its administration. Following adjustment of the medication regimen and anterior corpus callosotomy, an approximately 90% reduction in overall seizure burden was observed.

An ophthalmological assessment, requested as part of the postdiagnostic phenotypic characterization, documented intermittent exotropia without clinical nystagmus. Functional visual assessment was limited by the patient's level of cooperation. No lens opacities consistent with cataracts were identified. Fundus examination showed optic discs with preserved characteristics and no evident abnormalities of the macula or retinal vasculature, with no clinical evidence of optic atrophy.

Given the patient's baseline encephalopathic state, the palliative nature of the intervention, and the short postoperative follow-up period (94 days), the outcome was not classified using a definitive Engel scale category. Instead, the clinical response was described functionally by considering the reduction in overall seizure burden, the frequency of focal-to-bilateral tonic-clonic seizures, recurrence of status epilepticus, and the need for rescue medication (Table 1).

**Table 1:** Summary outcomes before and after callosotomy intervention ASM: anti-seizure medications

Variable	Pre-callosotomy	Post-callosotomy
Observation period	125 days, from December 2025 to April 2026	94 days, from April to July 2026
Seizure frequency	7-10 seizures/day on average; up to 15 seizures/day reported	First 24 h: no seizures. First postoperative week: one seizure reported, self-limited <5 min and without rescue medication. Later: two to threeseizures/week
Status epilepticus	Recurrent and super-refractory status epilepticus, with up to 10 episodes identified during the 125-day preoperative period	No postoperative status epilepticus reported during follow-up
Rescue medication use	Diazepam 10 mg IV, up to four rescue administrations/day. Recurrent need for anesthetic infusions for seizure control	One diazepam rescue dose only: 5 mg on May 2026; no further rescue doses documented
Continuous anesthetic therapy	Continuous midazolam infusion, approximately 0.35 mc/kg/h, and propofol 25 µg/kg/min for control of refractory ictal activity	Propofol discontinued postoperative day 2; midazolam discontinued postoperative day 6
Maintenance ASMs immediately before surgery	Valproate 1,200 mg q8h; topiramate 25 mg q12h; levetiracetam 1,500-1,000-1,500 mg/day; lacosamide 150-100 mg/day	Initially, the same maintenance ASM regimen immediately after surgery
Perioperative ASM changes	No meaningful maintenance ASM change immediately before surgery	No immediate perioperative maintenance ASM change
Subsequent postoperative ASM changes	-	Starting April: progressive increase of topiramate, initiation/titration of oxcarbazepine, gradual withdrawal of lacosamide; later valproate dose adjustments
Clinical response	Persistent high seizure burden despite polytherapy, frequent rescue treatment, and recurrent/super-refractory status epilepticus	Marked reduction in seizure burden, marked reduction in rescue medication requirement, successful withdrawal of continuous anesthetic therapy, and no recurrent status epilepticus
Discharge	-	June 2026; seizure frequency reportedly two to three times per week
ASMs at discharge	-	Topiramate 100 mg q12h; levetiracetam 1,500-1,000-1,500 mg/day; valproate 600 mg q8h; oxcarbazepine 300 mg q12h
Maximum postoperative follow-up available	-	94 days after surgery through July 2026

**Figure 3:**
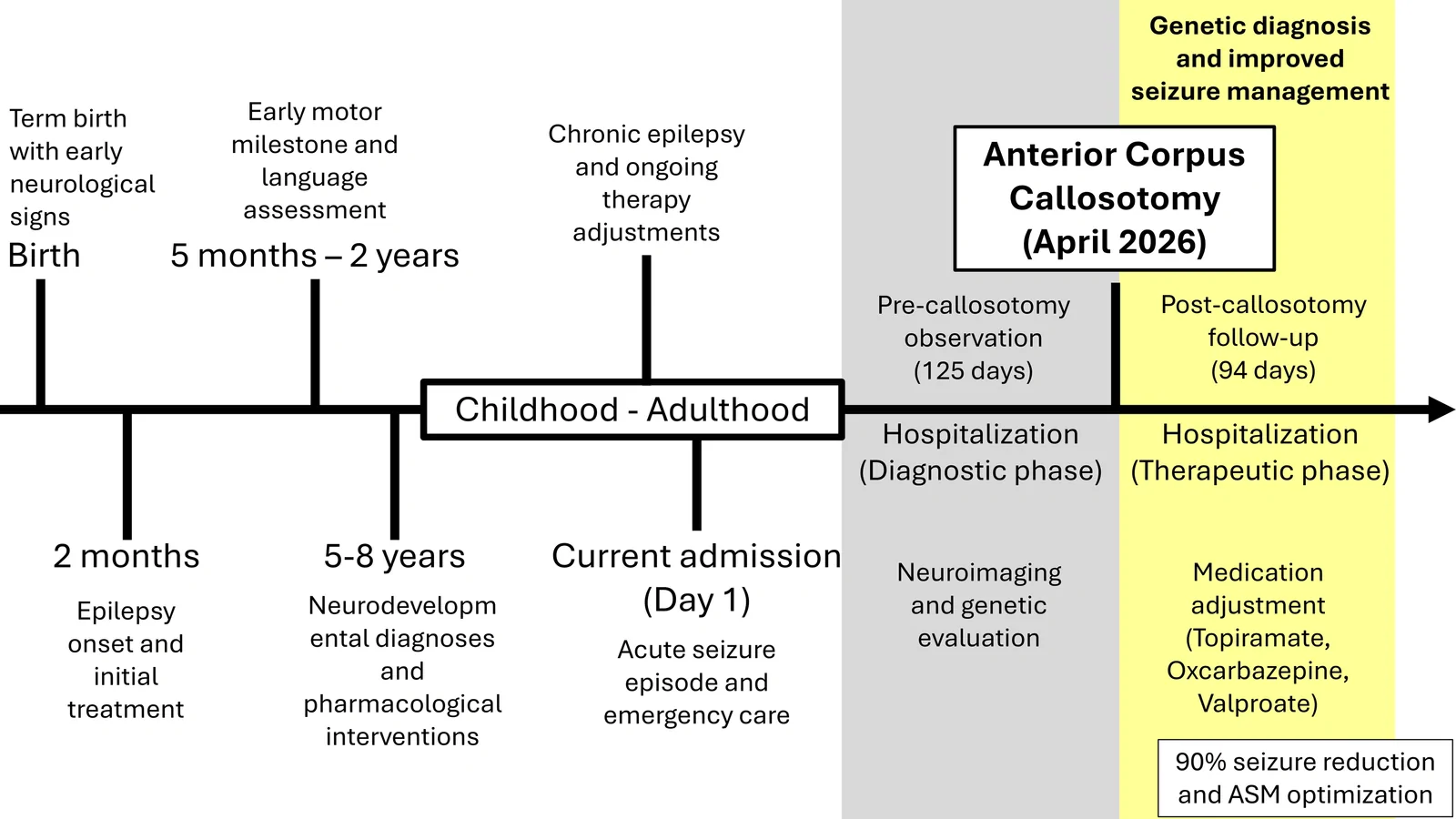
Timeline of the patient's 21-year diagnostic odyssey and clinical management. The schematic illustrates the progression from early developmental milestones and epilepsy onset to the chronic pharmacoresistant phase. The acute diagnostic and therapeutic interventions during the current admission are detailed, explicitly highlighting a 125-day pre-callosotomy observation period followed by a palliative anterior corpus callosotomy in April 2026. Subsequent therapeutic adjustments (including topiramate, oxcarbazepine, and valproate) and a 94-day post-callosotomy follow-up demonstrating an approximately 90% reduction in overall seizure burden are depicted. ASM: anti-seizure medications This image is an original, author-created schematic developed using Microsoft PowerPoint 2024 (Microsoft Corporation, Redmond, WA, USA).

A comprehensive timeline detailing the patient’s clinical milestones, chronic progression, and recent acute therapeutic intervention is summarized in Figure 3.

## Discussion

Biallelic TBC1D24 variants cause a spectrum of DEEs ranging from benign, self-limited forms to severe early-onset epileptic encephalopathies associated with profound intellectual disability and even premature death [9]. TBC1D24 alterations have also been reported in patients with isolated sensorineural hearing loss inherited in an autosomal recessive (OMIM #614617) or autosomal dominant (OMIM #616044) pattern; nevertheless, epilepsy remains the main clinical manifestation [6,7].

Epilepsy generally begins during the first year of life, at a median age of three months. Focal myoclonic seizures, with or without impaired awareness, are the most common seizure type and have been reported in up to 73% of patients. Status epilepticus is common, reaching a peak prevalence of 90% at 9-11 months of age. Drug-resistant seizures can be triggered by fever or infection and may occur in prolonged clusters. Movement disorders can also be presenting features, such as nonepileptic myoclonus (prevalence 100% between one and 17 months of age); tremor (prevalence 67% at 14 months); ataxia (prevalence 45% at 7.25 years); and episodic hemiplegia (prevalence 20% at 3.25 years [9,10].

Neurodevelopmental outcomes range from normal development without intellectual disability in benign forms to severe developmental delay and intellectual disability in severe forms. Some patients have congenital sensorineural hearing loss, particularly in the context of DOORS syndrome [9,11].

The human TBC1D24 gene encodes the protein of the same name, a vesicle-associated protein involved in neuritogenesis, neural crest cell and neuronal migration, neuronal maturation, and neurotransmission. In other words, TBC1D24 is a synaptic protein essential for neuronal vesicle trafficking [5,12,13]. Its described functions include regulation of presynaptic vesicle trafficking, maintenance of postsynaptic excitatory synapses, axonal development and neuronal growth, and interaction with KIBRA (kidney and brain protein) and Hippo signaling [9,12,14,15].

TBC1D24 plays a crucial role in synaptic vesicle trafficking and recycling at the presynaptic terminal; it is associated with clathrin-coated vesicles and synaptic sites in hippocampal neurons [12]. In addition, it is present at the postsynaptic sites of excitatory synapses and is required for dendritic spine maintenance through inhibition of the small GTPase ADP-ribosylation factor 6 (ARF6), a small GTP-binding protein involved in membrane trafficking between the plasma membrane and endocytic compartments [9,14].

TBC1D24 is a multifunctional protein consisting of 559 amino acids with two essential conserved domains: the N-terminal TBC domain and the C-terminal TLDc domain. The TBC domain is a member of the Rab GTPase-activating protein (Rab-GAP) family that regulates vesicle trafficking and recycling via modulation of Rab GTPase activity and its interaction with the small Ras-family GTPase ARF6 [9]. The TLDc domain may function as a sensor of reactive oxygen species that regulates vesicle-trafficking rates [5,9]. It also binds to the C2 domain of KIBRA, a scaffold protein encoded by WWC1 that functions in the Hippo signaling pathway and is important for human cognition and memory [16].

Variants in TBC1D24 cause endocytic dysfunction and disruption of the neuronal endosomal compartment, with decreased spontaneous neurotransmission. Haploinsufficiency in mice causes defective endocytosis, accumulation of enlarged endosomal compartments in neurons, and reduced spontaneous neurotransmission. In particular, TBC1D24 silencing in the adult mouse hippocampus causes dendritic spine loss, impaired contextual fear memory, hyperactivity, and increased anxiety [13,14].

Silencing TBC1D24 in primary rat cortical neurons causes defects in axonal specification, maturation of the axon initial segment, and action potential firing. Acute silencing in cerebrocortical neurons in vivo impairs callosal projections. Loss of TBC1D24 function induces membrane-trafficking abnormalities in the growth cone, leading to defects in axonal development and neuronal excitability [16].

Disease-associated variants affecting either conserved TBC1D24 domain are involved in neuronal development and survival and act predominantly as loss-of-function alleles [13,14]. Homozygous loss of TBC1D24 function has been suggested to correlate with a more severe prognosis; however, the broad clinical spectrum and range of variants preclude a clear genotype-phenotype correlation [17].

Variants in the TBC domain alter Rab-GAP function, impair presynaptic vesicle trafficking and recycling, and cause the accumulation of endosome-like vacuoles at synapses [18]. Variants in the TLDc domain disrupt its interaction with the KIBRA C2 domain. Indeed, two recessive epilepsy-associated variants, Gly511Arg and Ala515Val, have been reported to act through this pathogenic mechanism, linking the TBC1D24 and KIBRA pathways in epilepsy [15].

Furthermore, TBC1D24 contains a microexon that is incorporated postnatally. During embryonic and early postnatal development, the shorter TBC1D24 protein isoform, which excludes the microexon, is predominantly produced. Thus, the abrupt onset of seizures on postnatal day 15 correlates with a developmental shift toward microexon inclusion and may explain why some patients with variants affecting this microexon initially develop normally and subsequently experience an abrupt onset of epilepsy [12].

For clinical evaluation, the phenotypic spectrum can be categorized into benign phenotypes, including benign myoclonic epilepsy restricted to infancy, complete seizure control, and normal intellectual development; intermediate phenotypes, including FIME, early-onset drug-resistant epilepsy, and variable intellectual disability; severe phenotypes, including EIEE16, severe developmental delay, profound intellectual disability, premature death, and DOORS syndrome; and other reported phenotypes, including isolated hearing loss, parkinsonism, cerebellar ataxia, alternating hemiplegia of childhood, infantile-onset paroxysmal movement disorder, and multifocal polymyoclonus [9,19].

A diagnosis of TBC1D24-related epileptic encephalopathy is established by identifying biallelic pathogenic variants in TBC1D24, which are inherited in an autosomal recessive pattern [11]. Genetic testing should be considered in patients with early-onset myoclonic epilepsy, typically beginning during the first year of life; focal myoclonic seizures with or without impaired awareness; recurrent status epilepticus or prolonged seizures; congenital sensorineural hearing loss; developmental delay or intellectual disability; or movement disorders such as nonepileptic myoclonus, tremor, and ataxia [6,7,11].

TBC1D24 is associated with an unusually broad phenotypic spectrum, a diversity shared with only a few other epilepsy-related genes such as SCN1A, SCN2A, KCNQ2, KCNT1, PRRT2, and DEPDC5. This spectrum ranges from isolated neurological conditions, including benign myoclonic epilepsy, severe epileptic encephalopathy, and movement disorders like parkinsonism, cerebellar ataxia, and alternating hemiplegia, to multisystemic disorders like DOORS syndrome, and even nonepileptic conditions such as isolated hearing loss. Ancillary testing typically reveals nonspecific findings, including variable interictal EEG abnormalities without a pathognomonic pattern, inconsistent neuroimaging features, and generally normal metabolic profiles [9].

A recent longitudinal study comprising 197 patient-years of follow-up delineated the temporal evolution of these clinical features. Epileptic manifestations typically begin at a median age of three months, and status epilepticus is most common between nine and 11 months of age (90% prevalence). Seizure semiology is age-dependent, with the most prevalent presentation of focal seizures between 6.25 and 7.75 years of age (prevalence 88%) and myoclonic seizures between nine and 10 years of age (prevalence 80%). Concomitant movement disorders are present in a time-dependent manner. Nonepileptic myoclonus affects 100% of patients between one and 17 months of age, followed by tremor at 14 months (67%), episodic hemiplegia at 3.25 years (20%), and ataxia emerging later at 7.25 years (45%) [10].

Due to this variable clinical presentation and overall rarity, TBC1D24-related disorders pose substantial diagnostic challenges. The diagnostic odyssey typically involves multiple clinical evaluations, initial misdiagnoses, and considerable delays. Therefore, the implementation of NGS, WES, and WGS has transformed the diagnosis of TBC1D24-related disorders. WES is particularly effective as it can identify homozygous or compound-heterozygous pathogenic variants and detect cases of uniparental isodisomy (UPiD, through homozygosity mapping) and facilitates family segregation analysis using complementary Sanger sequencing to confirm parental carrier status, ultimately providing definitive molecular closure. Furthermore, WES allows the identification of cases that do not meet classic phenotypic descriptions, shortening the diagnostic odyssey, facilitating family genetic counseling, and providing access to clinical trials and emerging targeted therapies [11].

Most variants act as loss-of-function alleles. Seizures have been reported in heterozygous carriers, suggesting that haploinsufficiency may be clinically significant [13]. To date, intrafamilial phenotypic variability has not been reported among patients carrying the same variants [9].

Treatment of status epilepticus in TBC1D24-related disorders is generally based on standard escalation protocols in the acute setting, including early use of benzodiazepines, second-line anti-seizure medications such as valproate, levetiracetam, or fosphenytoin, and continuous infusion of anesthetics in refractory cases [20]. However, achieving long-term seizure control in these patients poses a unique pharmacological challenge. Regarding maintenance therapy specifically for TBC1D24-related disorders, phenobarbital has demonstrated high efficacy for acute and long-term seizure control; oxcarbazepine and topiramate reduce focal and myoclonic seizures [10].

Combination therapies have also proven successful in isolated reports, including high-dose phenobarbital with potassium bromide [11], oxcarbazepine with sulthiame, and valproate or phenobarbital in familial cases [9]. Agents such as everolimus, zonisamide, and the combination of phenytoin with clobazam have demonstrated favorable responses in individual patients [9]. Based on this emerging evidence, our patient's regimen was optimized with a combination of topiramate, oxcarbazepine, and valproate, deliberately avoiding phenobarbital as a maintenance option due to his previous episode of respiratory suppression.

Preventive strategies and home management have also been described. Home rescue therapy should be individualized, particularly for children with a history of recurrent status epilepticus. Early rescue protocols may include administering rescue medication at the onset of a convulsive seizure in patients with a history of recurrent status epilepticus. Seizure triggers should also be considered: seizures are often precipitated by fever or infection, and aggressive management of these factors may reduce seizure frequency [9,21].

However, when medical treatment is ineffective, other management strategies must be considered. Corpus callosotomy is a palliative surgical procedure for drug-resistant epilepsy, in which the cerebral hemispheres are disconnected to prevent interhemispheric spread of epileptic discharges and seizure generalization. Approximately 55% of patients achieve freedom from drop attacks, and 19% achieve complete seizure freedom after the procedure [22,23].

Since the introduction of NGS, DEE management has moved toward precision pharmacology and targeted therapies, but this case emphasizes the crucial role of palliative epilepsy surgery in monogenic disorders. In the past, a genetic etiology was often considered a relative contraindication for surgery. However, recent evidence suggests that procedures such as corpus callosotomy can drastically reduce seizure burden in genetic DEEs characterized by rapid bilateral synchronization or recurrent convulsive status epilepticus, as observed in our patient. Similar favorable surgical outcomes have been reported in other monogenic epilepsies, such as ATP1A3-related DEE and SCN8A-related epilepsy, demonstrating that the anatomical disconnection of epileptic networks is a highly effective synergistic strategy when precision medical therapy is insufficient to control life-threatening clinical exacerbations [8,24].

The TBC1D24 c.845C>G variant reported in this case is a transversion in which cytosine at position 845 of the coding DNA sequence is replaced by guanine, resulting in the amino acid substitution p.Pro282Arg (proline to arginine at position 282). This variant is located outside the two conserved functional domains of the TBC1D24 protein: the TBC domain, spanning amino acids 42-259, and the TLDc domain, spanning amino acids 342-554 [25].

Position 282 lies in the interdomain region between the N-terminal TBC domain and the C-terminal TLDc domain. This linker region may be important for the protein's structural stability or for specific protein-protein interactions. At the time of writing, this variant had been reported in patients of Mexican ancestry, and a possible founder effect had been suggested. Reported patients carrying this variant had refractory epilepsy, focal status epilepticus during infancy, substantial developmental delay, effort-induced movement disorders, and varying degrees of visual impairment [10]. These clinical features resemble those observed in our patient. Moreover, because he had failed to respond adequately to multiple antiseizure medications and had drug-resistant epilepsy not amenable to curative resective surgery, he underwent corpus callosotomy, with a favorable response.

This condition affects health in all three of its dimensions and has consequences for both patients and their families. Strategies to shorten the diagnostic odyssey are therefore essential. Early clinical suspicion should be raised in patients with myoclonic epilepsy beginning during the first year of life, particularly when accompanied by prolonged seizure clusters; seizures triggered by fever or infection; frequent episodes of status epilepticus or prolonged seizures; early drug resistance; epilepsy combined with hearing loss, with or without other features of DOORS syndrome; or associated movement disorders such as nonepileptic myoclonus, tremor, ataxia, or episodic hemiplegia [19].

Early diagnosis is fundamental to the natural history and management of the disease. For patients and their families, it marks the end of the diagnostic odyssey, reduces unnecessary invasive testing, enables accurate genetic counseling regarding the 25% recurrence risk associated with autosomal recessive inheritance, provides access to condition-specific support groups and resources, and informs eligibility for clinical trials. From a clinical management perspective, it may help optimize evidence-based antiseizure therapy - phenobarbital, oxcarbazepine, topiramate, and everolimus have shown greater efficacy - while prompting early audiological assessment, neurodevelopmental monitoring, evaluation for features of DOORS syndrome when present, and planning of multidisciplinary interventions [10].

## Conclusions

We report the case of a Mexican male patient with DEE due to a homozygous variant of TBC1D24, who experienced a 21-year diagnostic odyssey and had a good response to the adjustment of the medication regimen and the corpus callosotomy as part of multimodal treatment. The basis for follow-up of patients is a multidisciplinary approach, especially in patients with difficult-to-control clinical manifestations and with a family history of similar disorders.

The diagnosis and treatment of this patient were possible through the collaboration between the neurology, neurosurgery, and medical genetics departments, and the entire multidisciplinary team involved in the patient’s evaluation.
